# Quantitative Evaluation of the Substantially Variable Morphology and Function of the Left Atrial Appendage and Its Relation with Adjacent Structures

**DOI:** 10.1371/journal.pone.0126818

**Published:** 2015-07-31

**Authors:** Cai-Ying Li, Bu-Lang Gao, Xiao-Wei Liu, Qiong-Ying Fan, Xue-Jing Zhang, Guo-Chao Liu, Hai-Qing Yang, Ping-Yong Feng, Yong Wang, Peng Song

**Affiliations:** 1 Department of Medical Imaging, Second Hospital, Hebei Medical University, Shijiazhuang, Hebei Province, PR China; 2 Department of Medical Research, Shijiazhuang First Hospital, Hebei Medical University, Shijiazhuang, Hebei Province, PR China; University of Bologna, ITALY

## Abstract

**Objective:**

To investigate quantitatively the morphology, anatomy and function of the left atrial appendage (LAA) and its relation with adjacent structures.

**Materials and Methods:**

A total of 860 patients (533 men, 62.0%, age 55.9±10.4 year) who had cardiac multidetector computed tomography angiography from May to October 2012 were enrolled for analysis.

**Results:**

Seven types and 6 subtypes of LAA morphology were found with Type 2 being the most prevalent. Type 5 was more significantly (P<0.05) present in women (8.0%) than in men (4.2%). LAA orifice was oval in 81.5%, triangular in 7.3%, semicircular in 4%, water drop-like in 3.2%, round in 2.4% and foot-like in 1.6%. The LAA orifice had a significantly greater (P<0.01) major axis in men (24.79±3.81) than in women (22.68±4.07). The LAA orifice long axis was significantly (P<0.05) positively correlated with the height, weight and surface area of the patient. The LAA morphology parameters displayed strong positive correlation with the left atrium volume, aortic cross area long axis or LSPV long axis but poor correlation with the height, weight, surface area and vertebral body height of the patients. Four types of LAA ridge were identified: AI, AII, B and C with the distribution of 17.6%, 69.9%, 5.9% and 6.6%, respectively. The LAA had a significantly (P<0.05) greater distance from its orifice to the mitral ring in women than in men. The LAA had two filling and two emptying processes with the greatest volume at 45% phase but the least volume at 5% phase. The LAA maximal, minimal and emptying volumes were all significantly (P<0.05) positively correlated with the body height, weight and surface area, whereas the LAA ejection fraction had an inverse correlation with the LAA minimal volume but no correlation with the maximal volume.

**Conclusion:**

The LAA has substantially variable morphologies and relation with the adjacent structures, which may be helpful in guiding the LAA trans-catheter occlusion or catheter ablation procedures.

## Introduction

Cardiac arrhythmias comprise an important public health problem significantly associated with elevated risks of sudden death and cardiovascular complications, subsequently leading to decreased quality of life, disability, high mortality and massive healthcare expenses[1–3]. Atrial fibrillation is the most common sustained cardiac arrhythmia encountered in daily clinical practice, affects over 2.3 million people in the United States and has been increasingly on the rise with ageing of the society[2, 3]. The current prevalence of atrial fibrillation in the general population is 0.5%-1% and goes up to approximately 10% for those who are 80 years of age or above in the Western countries[4–7]. Data from some Asian countries including China showed a lower prevalence of atrial fibrillation (0.2%-1.5%)[3, 8]. However, since Asia is the most populated area in the world, the burden of atrial fibrillation in Asia may actually exceed that in Western countries. Atrial fibrillation carries a significantly increased risk of stroke, and the risk of stroke rises approximately 5-fold in non-rheumatic atrial fibrillation and 17-fold in patients with mitral stenosis and atrial fibrillation[7, 9]. Around 15% of ischemic strokes are caused by atrial fibrillation, and 90% of atrial thrombi in non-rheumatic atrial fibrillation and 60% of such thrombi in patients with rheumatic mitral valve stenotic diseases originate from within the left atrial appendage (LAA)[9, 10]. Cardioembolic stroke is the most serious and life threatening potential complication of atrial fibrillation, with an associated mortality up to 30% at 12 months and a 1/3 recurrence rate at 5 years [11, 12]. Stroke prevention thus plays a socio-economically highly important role in atrial fibrillation management.

Currently, the most established prophylaxis for stroke in atrial fibrillation is the dose-modulated oral anticoagulation with vitamin-K-antagonists, with warfarin being the most widely investigated drug[11]. Of note, it is often difficult to maintain long-term anticoagulation within a narrow therapeutic range partly because of regular laboratory monitoring of anticoagulation intensity, frequent dose adjustment and higher risks of bleeding during anticoagulation [13, 14]. Patients with atrial fibrillation, high risk of stroke and contraindications to long-term oral administration of anticoagulants due to hemorrhage or other secondary adverse effects may be candidates for alternative treatment that combines high efficacy in stroke prevention and low hemorrhage risk. The frequency of thrombus formation in LAA and the dominant role of LAA as a source of embolism in patients with atrial fibrillation led to the hypothesis that resection or obliteration of the LAA might decrease the risk of stroke [15, 16]. However, due to the invasive nature of surgery, surgical elimination of LAA is only performed as an adjunctive procedure in patients undergoing mitral valve surgery[17]. Moreover, surgical LAA exclusion is incomplete in 1/3 to 1/2 patients[16, 18]. Thus, the development of less invasive percutaneous approaches to completely obliterate the LAA via trans-catheter delivery of a mechanical device is a great step ahead in this field, and this catheter-delivered mechanical device may allow for direct prevention of strokes associated with LAA thromboembolism. Such a device has to be a reasonable size and shape matching a particular patient’s LAA anatomy or be able to adapt to a variety of LAA dimensions in order to completely occlude and be safely retained within the LAA. Therefore, it is extremely important to have a good understanding of the LAA morphology, anatomic variations and functions present in patients with atrial fibrillation. Drug-refractory atrial fibrillation can also be treated with catheter-based radiofrequency ablation, however, this complex electrophysiological procedure also requires a good understanding of the anatomy and morphology of the LAA and the adjacent structures such as the pulmonary veins, left atrium roof and coronary arteries for increasing successful ablation outcome and avoiding potential complications[19–21]. This study was to investigate quantitatively the anatomy, morphology and function of the LAA and its relationship with the adjacent structures which have not been clearly stated in the literature.

## Materials and Methods

### Subjects

A total of 860 patients (533 men, 62.0%, and 327 women, 38.0%; age range: 22–88 years, mean: 55.9±10.4) who had cardiac multidetector (256 slices) computed tomography (MDCT) angiography from May 1st to October 2012 were enrolled in this study. The Ethics Committee of the Second Hospital and Shijiazhuang First Hospital, Hebei Medical University approved this study and signed consent was obtained from all patients. The indications for MDCT coronary angiography included atypical chest pain, angina pectoris, symptoms of coronary heart disease, difficulty in diagnosis based on conventional angiography, follow-up after drug treatment for coronary heart disease, coronary artery stenting or coronary artery bypass grafting, and screening for coronary heart diseases in patients with hypertension, diabetes mellitus and hyperlipemia or before cardiac surgery. Contraindications were pregnancy, respiratory or hepatic insufficiency, poor renal function (serum creatinine >1.5 mg/dl), known allergic reaction to contrast media, poor clinical condition, severe arrhythmia or tachycardia.

Among the original 860 patients, the LAA morphology was analyzed in 670 patients (male 433, 64.6%; female 237, 35.4%; age range 22–88 year, mean 55.9±10.4). Quantitative measurement of the LAA volume and depth, orifice shape and axis, and relation with adjacent structures was performed in 150 people (male 80, female 70; age range 28–70 year, mean 50.5±9.1) who had normal MDCT results with normal sinus rhythm and blood pressure excluding atrial fibrillation, valvular heart disease, congenital heart disease or cardiomyopathy. The LAA and left atrium volumes and function were quantitatively evaluated in 40 normal people (male 20, female 20, age range 28–78 year, mean 50.5±9.6) who had normal MDCT results with normal sinus rhythm and blood pressure excluding atrial fibrillation, valvular heart disease, congenital heart disease or cardiomyopathy.

### MDCT scan protocol and image analysis

The ECG-gating MDCT coronary angiography was performed with a 256-slice CT scanner (Brilliance iCT, Philips Healthcare, Cleveland, OH, USA) during a breath hold of 4–7 seconds. The scan parameters were as follows: tube voltage 80–120 kV, tube current 280–350 mAs, detector collimation 128×0.625 mm, pitch 0.18, gantry rotation time 330 ms, matrix 512 ×512, and field of view 250 mm. The contrast medium iohexol (1.0 mL/kg or 350 mgl/mL) was intravenously injected at a flow rate of 4–5 mL/s into the antecubital vein through a single-tube high pressure syringe. The MDCT coronary angiogrpahic scanning was started from the tracheal bifurcation to the superior border of the liver with the ECG-trigger level set in the descending aorta at the level of pulmonary artery.

The raw images were reconstructed at the 75% R-R interval and then transferred to the Philips EBW 4.5 workstation (Extended Brilliance Workspace, V4.5.2.4031, Philips Healthcare Nederland B.V., The Netherlands) for further analysis with specialized software (Vitrea 2; Vital Images, Inc., Minneapolis, MN, USA). For LAA volume and function test, ten phases (5%-95%, interval 10%) over one cardiac cycle were extracted and transferred to specialized software (Philips EBW 4.5 workstation, The Netherlands) for LAA reconstruction. Besides traditional axial images, three-dimensional volume-rendering images, multiplanar reconstructed images and virtual endoscopy were used to evaluate the LAA anatomical shape variations. All images were reviewed by two experienced radiologists (more than 20 years of experience) working independently. The types of the LAA morphology and the left atrial orifice shape were evaluated carefully by the two observers, and if different opinions arose regarding the type definition, a third observer would be involved to judge the definition of the morphology. The total LAA emptying volume (LAAEV) was calculated as the difference between maximal (LAAVmax) and minimal (LAAVmin) LAA volumes (LAAEV = LAAVmax-LAAVmin). The LAA ejection fraction (LAAEF) was calculated: LAAEF = (LAAVmax-LAAVmin)/LAAVmax.

### Statistical analysis

All data were statistically processed with SPSS 17.0 software. Chi-square testing was used for analysis of gender-specific distribution of the LAA. A level of P < 0.05 was considered statistically significant. Continuous data were expressed as mean ± SD.

## Results

### LAA anatomical morphology variations

**Fig 1:**
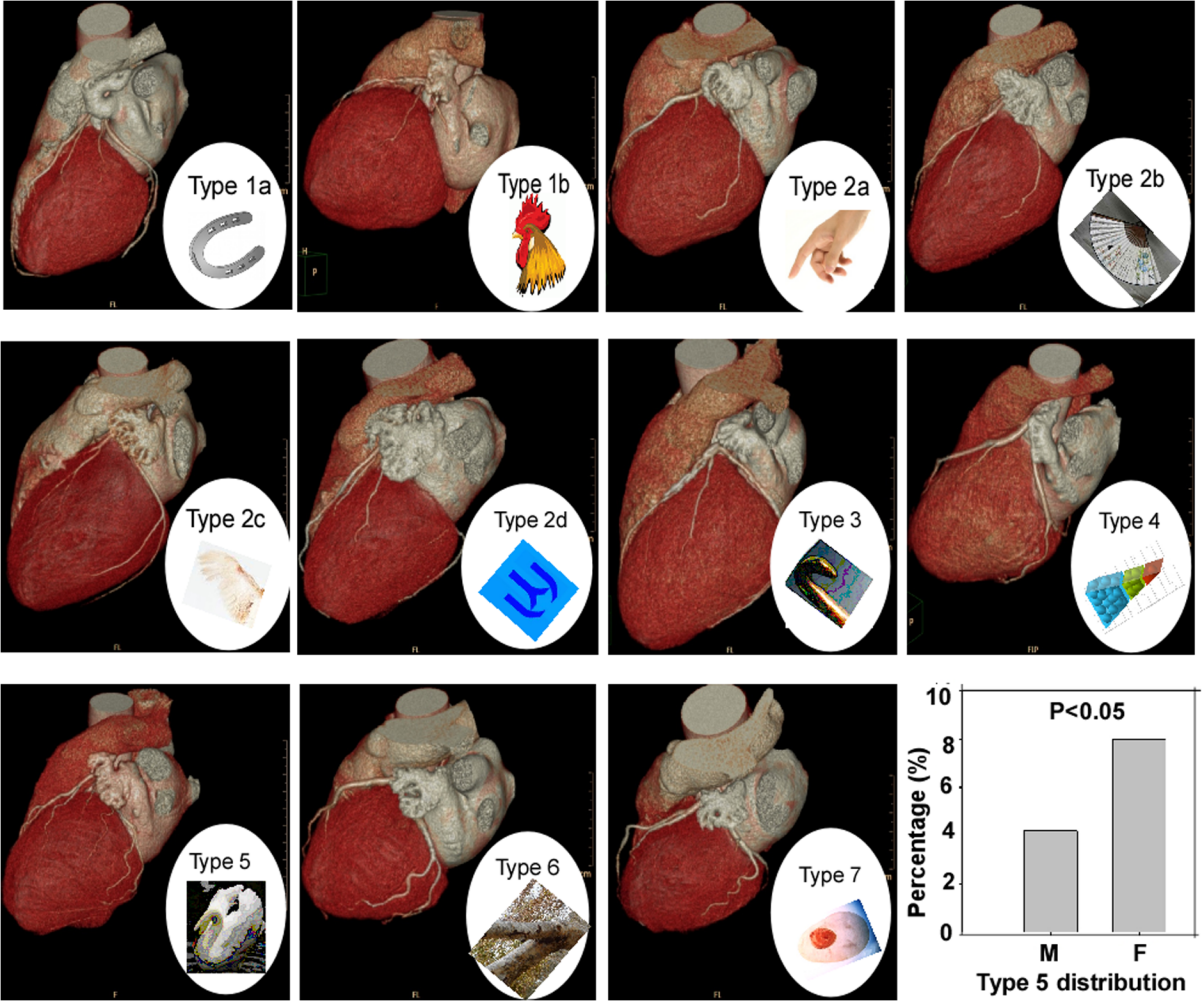
Seven types with 6 subtypes of the left atrial appendage (LAA) based on LAA tip orientation and anatomical shape. Type1a: horseshoe, Type1b: cockscomb, Type2a: hand finger, Type2b: fan, Type2c: wing, Type2d: mountain like the Chinese character “山”, Type3: hook, Type4: wedge, Type5: swan, Type6: fork, Type7: ring. T, type; LSPV, left superior pulmonary vein; LIPV, left inferior pulmonary vein; MPA, main pulmonary artery. Type 5 was more significantly (P<0.05) frequently present in women (8.0%) than in men (4.2%).

**Fig 2:**
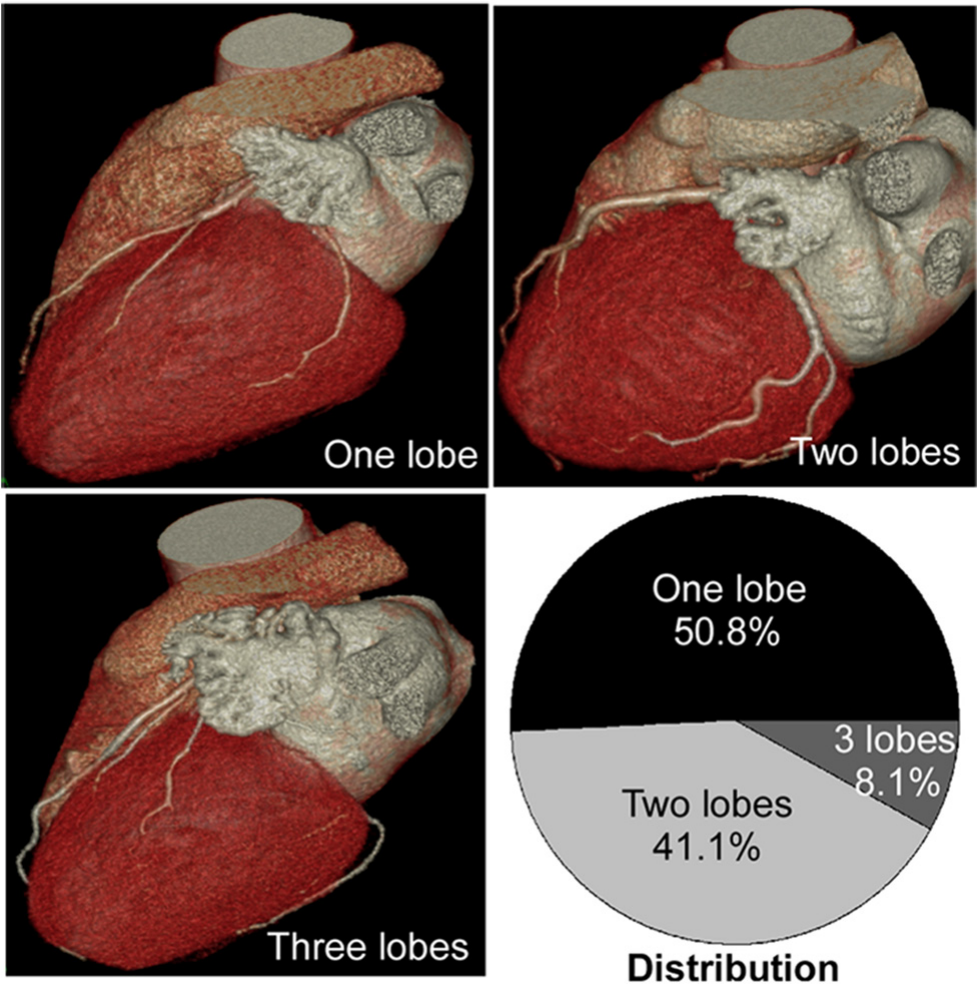
Three-dimensional volume-rendering images showed the number of left atrial appendage lobes and their distribution.

Analysis of the 670 consecutive patients with cardiac MDCTA revealed 7 types and 6 subtypes of LAA morphology (Table 1 and Fig 1). The incidence of types 1–7 in the 670 patients was 6.2%, 76.6%, 3.1%, 4.0%, 5.5%, 3.7% and 0.7%, respectively. Type 2 was the most prevalent followed by types 1 and 5. Type 2c was also the most prevalent among all the subtypes of type 2 while type 7 had the lowest prevalence. Type 5 was more significantly (P<0.05) present in women (8.0%) than in men (4.2%) (Table 2 and Fig 1), but no statistically significant (P>0.05) prevalence existed in other types between men and women. LAA lobe was defined as an outpouching of the LAA of at least 1 cm in width and depth according to the criteria described by Budge et al[22]. One to three lobes were identified in LAA, with 1 lobe in 50.8%, 2 lobes in 41.1% and 3 lobes in 8.1% (Fig 2). No LAA with 4 lobes or more was found.

**Table 1:** Left atrial appendage (LAA) anatomical classification.

Classification	Name	Description
Type 1a	Horseshoe	LAA tip is like a horseshoe to orient upward, parallel to MPA
Type 1b	Cockscomb	LAA tip is oriented upward and then extends forward and backward to form a cockscomb
Type 2a	Hand finger	Long LAA tip is like a finger oriented downward, parallel to MPA
Type 2b	Paper fan	Short LAA tip is like a paper fan oriented downward, parallel to MPA
Type 2c	Wing	LAA tip is like a chicken wing oriented downward, parallel to MPA
Type 2d	Mountain	LAA tip has 3 main tips with long downward tips parallel to MPA like a mountain
Type 3	Hook	LAA tip is oriented upward but then turns medially between MPA and LA to form a hook
Type 4	Wedge	LAA tip is wedge like to orient upward and then backward
Type 5	Swan	LAA tip is oriented upward and then forward, parallel to MPA, to form a swan
Type 6	Fork	LAA has two main tips oriented in different directions to form a fork
Type 7	Ring	LAA has a ring structure with no tip

Note: MPA, main pulmonary artery; LA, left atrium

**Table 2:** Distribution of left atrial appendage types in males versus females.

Types	Males	Females	Total
Type 1a	25 (5.8%)	8 (3.4%)	33 (4.9%)
Type 1b	5 (1.2%)	4 (1.7%)	9 (1.3%)
Type 2a	40 (9.2%)	25 (10.5%)	65 (9.7%)
Type 2b	102 (23.6%)	44 (18.6%)	146 (21.8%)
Type 2c	163 (37.6%)	94 (39.7%)	257(38.4%)
Type 2d	30 (6.9%)	15 (6.3%)	45 (6.7%)
Type 3	13 (3.0%)	8 (3.4%)	21 (3.1%)
Type 4	18 (4.2%)	9 (3.8%)	27 (4.0%)
Type 5	18 (4.2%)	19 (8.0%)*	37 (5.5%)
Type 6	16 (3.7%)	9 (3.8%)	25 (3.7%)
Type 7	3 (0.7%)	2 (0.8%)	5 (0.7%)
Total	433(64.6%)	237(35.4%)	670(100%)

* P = 0.036 compared with the males in type 5.

### Quantitative evaluation of the LAA anatomy

**Fig 3:**
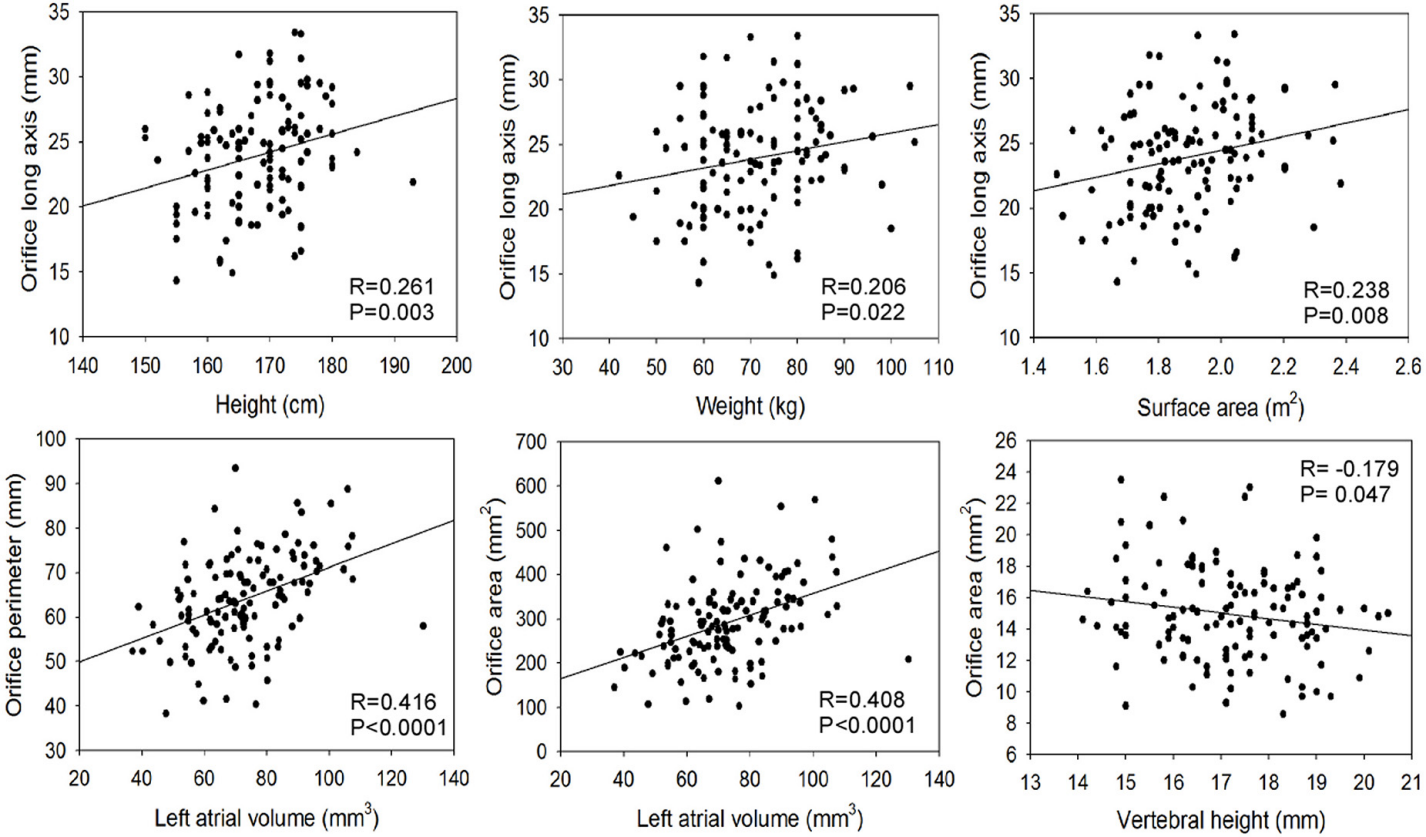
Correlation of the left atrial appendage (LAA) morphology with other parameters. The LAA orifice long axis is positively correlated with the height, weight and body surface area. The first curve angle of the LAA is positively correlated with the weight of the body. The aortic long axis of its cross area is positively correlated with LAA orifice short and long axes, perimeter, area and volume. The left atrial volume positively correlates with the LAA orifice short and long axes, perimeter and area, LAA depth and volume, and the first curve angle. The left superior pulmonary vein (LSPV) long axis correlates positively with the LAA orifice short and long axes, perimeter and area, and the LAA volume, whereas the vertebral body height correlates negatively with the LAA orifice area.

Quantitative measurement of the LAA anatomy was performed in 150 normal people. The shape of the LAA orifice, defined as the site of reflection of this structure with the surrounding left atrial wall, was oval in 81.5%, triangular in 7.3%, semicircular in 4%, water drop-like in 3.2%, round in 2.4% and foot-like in 1.6%. The LAA orifice had a significantly greater (P<0.01) major axis in men (24.79±3.81) than in women (22.68±4.07) and no other significant difference (P>0.05) existed in the gender distribution (Table 3). The LAA morphology parameters did not correlate with age (Table 3). However, the LAA orifice long axis was significantly (P<0.05) positively correlated with the height (R = 0.0261, P = 0.003), weight (R = 0.206, P = 0.022) and surface area (R = 0.238, P = 0.008) of the patients while the orifice short axis showed negative correlation with the above parameters without significance (P>0.05) (Fig 3, Table 4 and S 1–3). The LAA orifice short and long axes, perimeter, area and the LAA volume were significantly (P<0.05) positively correlated with the aortic cross area long axis, the left atrial volume and the LSPV cross area long axis, respectively (Table 4 and Fig 3). The left atrial volume was also significantly (P<0.05) positively correlated with the LAA depth and the first curve angle of the LAA. However, the vertebral height was significantly (P<0.05) negatively correlated with the LAA orifice area. The LAA morphology parameters (orifice short and long axes, perimeter, area, and the LAA volume) displayed significantly (P<0.05) strong positive correlation with the left atrium volume, aortic cross area long axis or LSPV long axis but poor correlation with the height, weight, surface area and vertebral body height of the patients (Fig 3 and Table 4).

**Table 3:** LAA morphology reference range and comparison between different genders and ages ($\bar{x}$±s).

Parameters	Range	95% Range ($\bar{x}$±s)	Gender		Age (y)		
			Male(n = 80)	Female(n = 70)	<40(n = 26)	40–60(n = 85)	>60(n = 39)
Orifice long axis (mm)	14.3–33.4	15.30–31.86 (23.94±4.04)	24.79±3.81	22.68±4.07*	24.74±3.43	23.79±4.28	22.9±4.12
Orifice short axis (mm)	8.6–23.5	9.11–20.83 (14.97±2.99)	14.57±2.81	15.56±3.16	15.75±3.98	15.02±3.37	14.41±2.47
Orifice area (mm^2^)	104.1–610.6	89.05–447.91 (291.91±94.90)	291.34±91.31	292.75±100.92	306.11±71.93	290.42±101.97	273.52±78.44
Orifice perimeter (mm)	38.4–93.3	43.83–84.09 (63.96±10.27)	64.50±9.61	63.17±11.23	66.32±6.31	63.53±11.40	62.61±9.79
LAA depth (mm)	25.9–59.2	29.11–54.05 (41.58±6.36)	42.15±6.43	40.73±6.21	41.70±6.20	41.41±7.20	41.43±5.74
LAA volume (ml)	2.1–13.6	2.46–10.66 (6.56±2.09)	6.62±1.91	6.44±2.33	6.76±1.78	6.57±2.15	6.55±2.11
LAA 1st bend angle (°)	69–150	80.62~147.80 (114.21±17.14)	114.3±17.10	114.08±17.26	121.00±12.99	114.70±17.01	109.00±20.29

Note: LAA, left atrial appendage.

*P<0.01 compared with males in the orifice long axis. No significant difference (P>0.05) was found between different ages in each of the parameters.

**Table 4:** Correlation analysis between LAA morphology and other parameters (R values).

Patient baseline features	Orifice long axis	Orifice short axis	Orifice area	Orifice perimeter	LAA depth	LAA volume	LAA 1st bend angle (°)
Height	0.261^**^	-0.033	0.048	0.107	0.008	0.073	0.019
Weight	0.206^*^	-0.089	0.053	0.106	0.057	0.128	0.178^*^
Surface area	0.238^**^	-0.083	0.056	0.115	0.049	0.125	0.153
Aortic cross area major axis	0.225^*^	0.231^**^	0.268^**^	0.260^**^	-0.056	0.291^**^	0.051
Vertebral body height	0.175	-0.179^*^	-0.071	0.001	-0.033	-0.010	-0.055
LSPV long axis	0.463^**^	0.194^*^	0.329^**^	0.402^**^	0.081	0.358^**^	-0.042
LA volume	0.381^**^	0.325^**^	0.408^**^	0.416^**^	0.187^*^	0.450^**^	0.178^*^

Note:*P<0.05

**P<0.01. LA, left atrium; LAA, left atrial appendage

### LAA relation with adjacent structures

**Fig 4:**
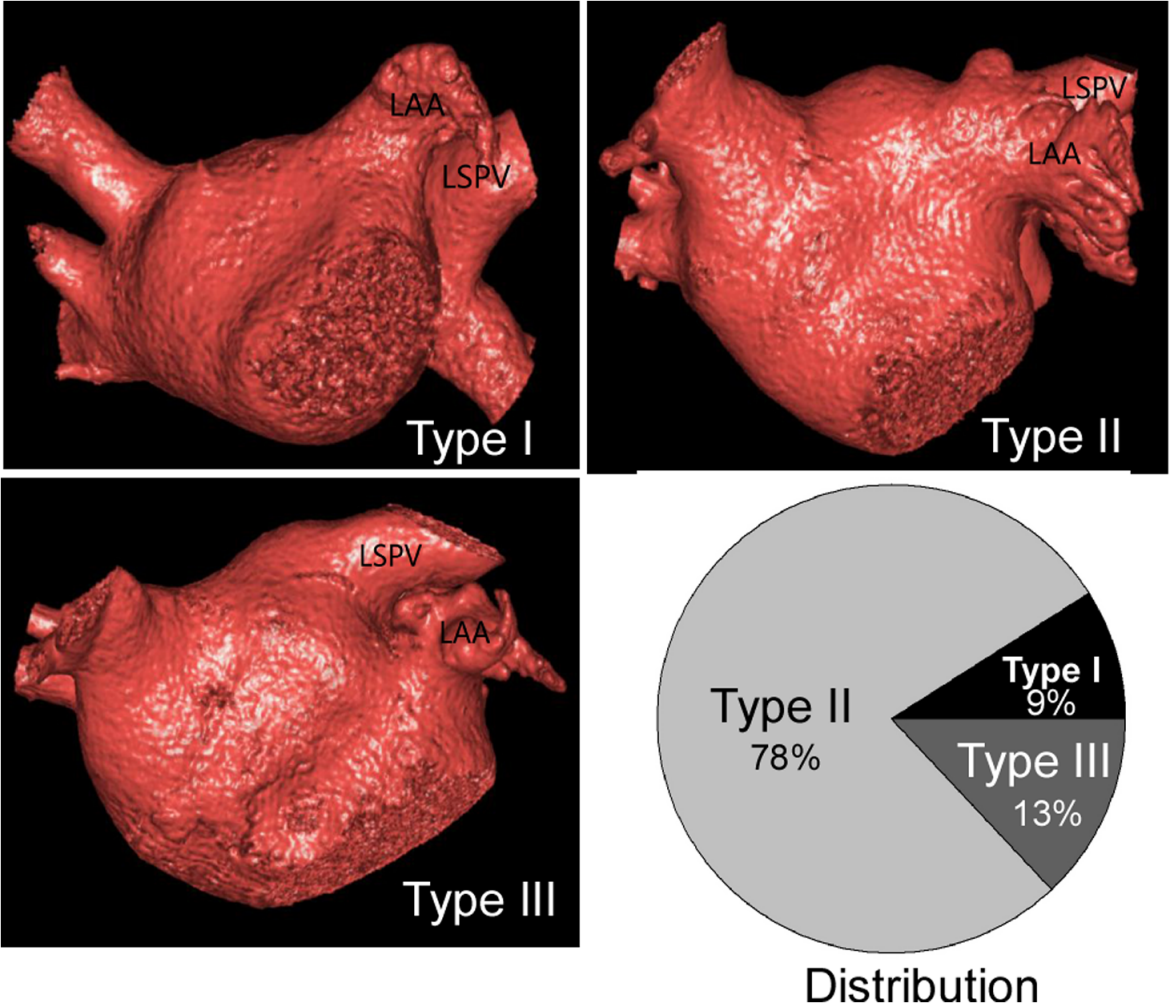
Three-dimensional images showed the three types of the left atrial appendage (LAA) based on its relationship with the left superior pulmonary vein (LSPV) and left inferior pulmonary (LIPV) vein: Type I, the LAA is superior to the LSPV; Type II, the LAA is horizontal to the LSPV; Type III, the LAA is inferior to the LSPV. The distribution of different types of LAA was also shown.

**Fig 5:**
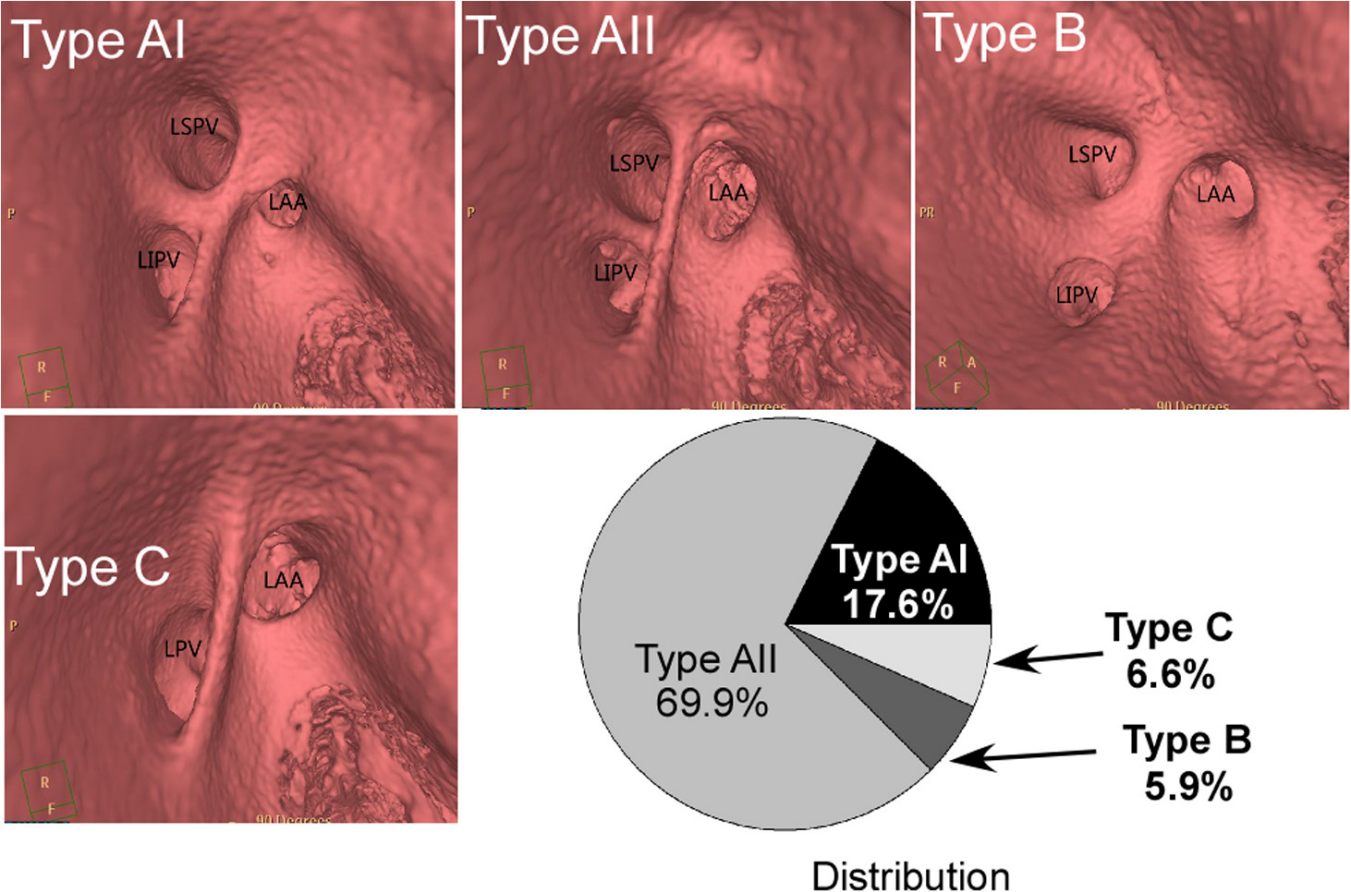
Virtual endoscopic view of the left atrial appendage (LAA) ridge types: AI, AⅡ, B and C and their distribution. Type A I: the LAA ridge extends from the superior portion of the left superior pulmonary vein (LSPV) orifice to the inferior portion of the left inferior pulmonary vein (LIPV) orifice on the same plane with the intervenous saddle between the LSPV and the LIPV. Type A II: the LAA ridge is markedly prominent above the intervenous saddle between the LSPV and LIPV and extends from the superior portion of the LSPV orifice to the inferior portion of the LIPV orifice. Type B: the ridge extends from the superior portion of the LSPV orifice to the intervenous saddle between the LSPV and LIPV orifices. Type C: the LSPV and the LIPV have a common venous trunk and the LAA ridge runs from the upper edge of the trunk to the lower edge. Type A II (69.9%) accounts for the greatest distribution of all cases followed by types A I (17.6%), C (6.6%) and B (5.9%).

**Fig 6:**
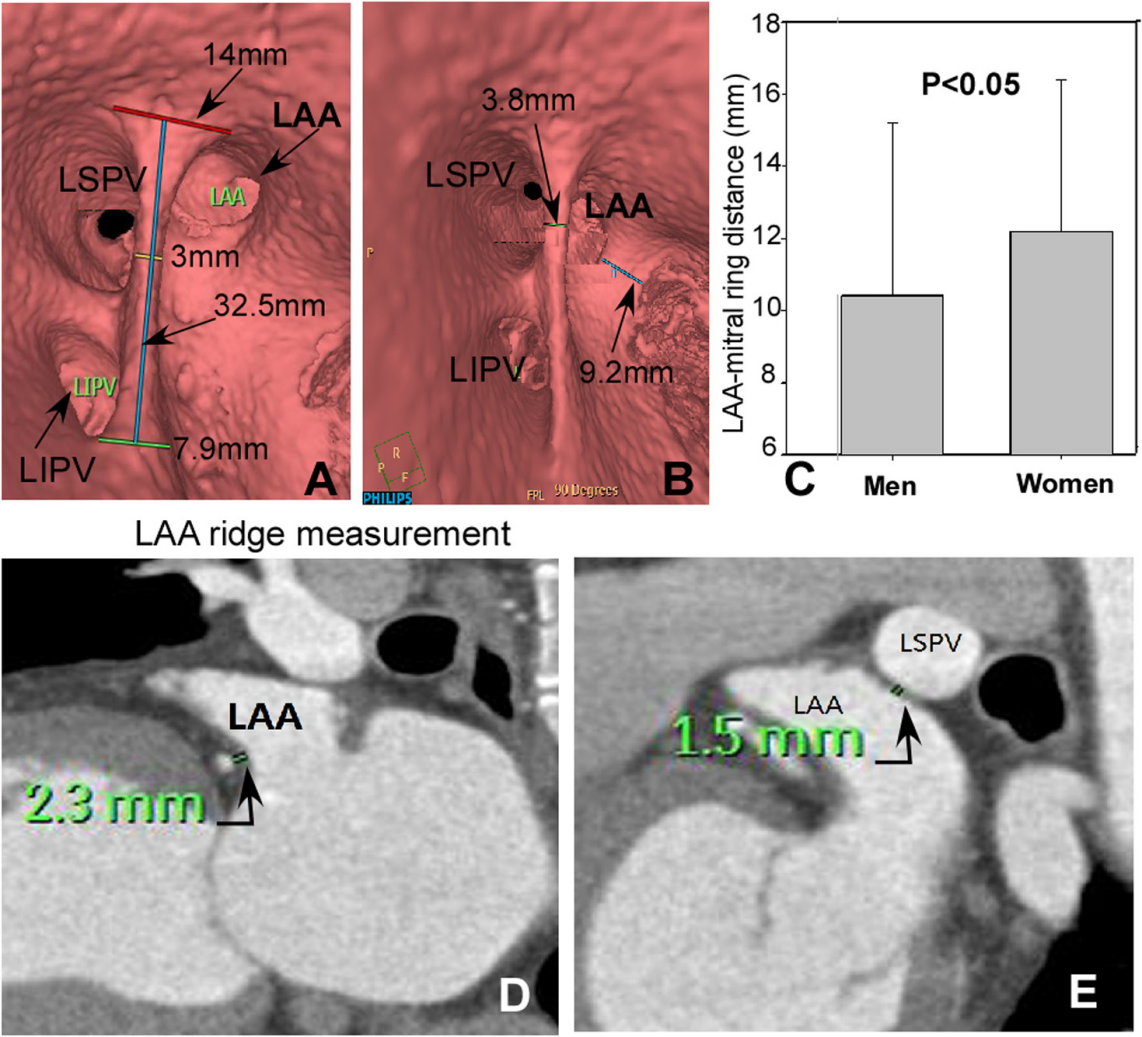
Virtual endoscopic view of left atrial appendage (LAA) ridge. A: The length of LAA ridge (blue line), the length of the superior border of LAA ridge (red line), the width of the LAA ridge middle part (yellow line), and the length of LAA ridge inferior margin (green line). B: The distance from the LAA ostium to the left superior pulmonary vein (LSPV) ostium (green line) and the distance between LAA ostium and mitral annulus (blue line). C. The LAA had a significantly (P<0.05) greater distance from its orifice to the mitral ring in women than in men. D. The minimal distance from LAA to left circumflex artery (green line) was indicated. E. The minimal distance from LAA to LSPV (green line) was shown. LIPV: left inferior pulmonary vein.

There were three types of LAA orifice based on its relative position with the orifice of the LSPV (Fig 4) and the distribution was 9% (14 cases), 78% (117 cases) and 13% (19 cases) for types 1, 2 and 3, respectively. The LAA ridge is prominent and interposed between the LAA and the LSPV orifices. Four types of LAA ridge were identified (Fig 5): types AI, AII, B and C with the distribution of 17.6% (n = 26), 69.9% (n = 105), 5.9% (n = 9) and 6.6% (n = 10), respectively. No significant difference (P>0.05) existed in either the length or width of LAA ridge between men and women (Fig 6 and Table 5). The LAA had a significantly (P<0.05) greater distance from its orifice to the mitral ring in women than in men (Fig 6C), but no significant differences (P>0.05) in other parameters were detected between men and women.

**Table 5:** Relationship of LAA with adjacent structures.

Parameters	Range ($\bar{\text{x}}$±s, mm)	Males (n = 80, mm)	Females (n = 70, mm)
D(LAA orifice-LSPV orifice)	2.5–11.5(4.9±1.7)	4.9±1.7	4.9±1.7
Dmin (LAA-LSPV)	0.5–5.4(1.3±0.9)	1.1±0.7	1.47±1.1
D (LAA-mitral ring)	5.5–18.2(10.6±2.3)	10.2±2.4*	11.1±2.1*
D (LAA-LCA)	1.0–6.6(2.1±0.9)	2.01±0.8	2.2±0.9
Ridge length	18.1–44.0(33.2±5.2)	33.8±5.5	32.9±4.7
Upper ridge width	4.0–19.9(11.0±3.2)	10.7±3.0	11.4±3.5
Middle ridge width	1.7–7.6(4.0±1.1)	3.9±1.0	4.1±1.2
Lower ridge width	3.8–15.9(7.4±2.2)	7.6±2.2	7.0±2.1

Note: LAA, left atrial appendage; D, distance; LSPV, left superior pulmonary vein; LCX, left circumflex artery; Dmin, the minimum distance. D(LAA orifice-LSPV orifice): The distance from LAA orifice to LSPV orifice; D(LAA-LSPV) min: The minimum distance from LAA to LSPV; D(LAA-mitral ring):The distance between LAA orifice and mitral ring; D(LAA-LCA):The minimum distance from LAA to left circumflex artery.

* indicates a significant (P<0.05) difference between males and females.

### Evaluation of LAA volume and function

**Fig 7:**
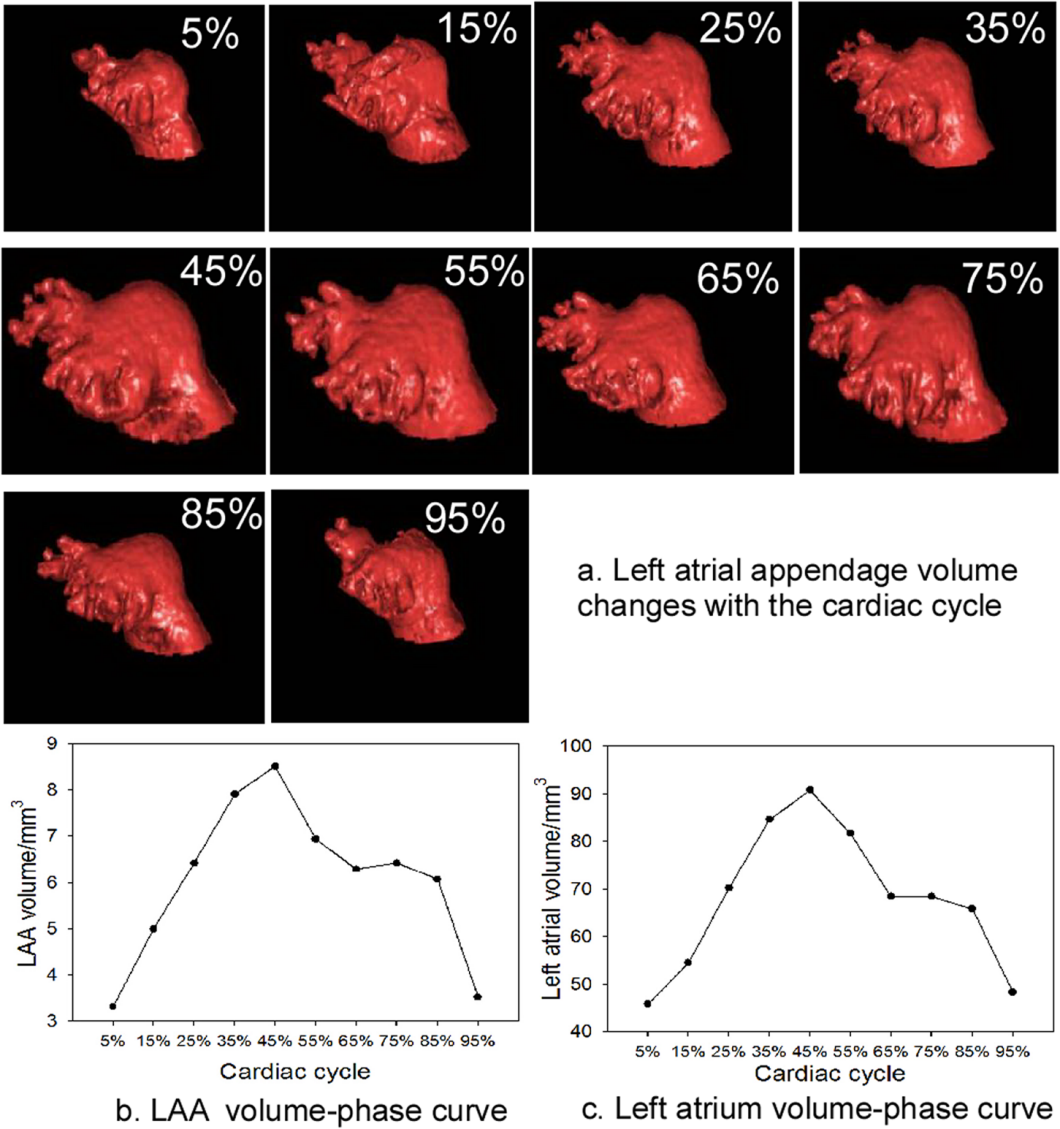
The volumes of the left atrial appendage (LAA) and the left atrium change at the cardiac cycle (5% phase-95% phase). a. The LAA volume changes with the cardiac cycle. b. The LAA volume-phase curve at the cardiac cycle. c. The left atrium volume-phase curve at the cardiac cycle.

**Fig 8:**
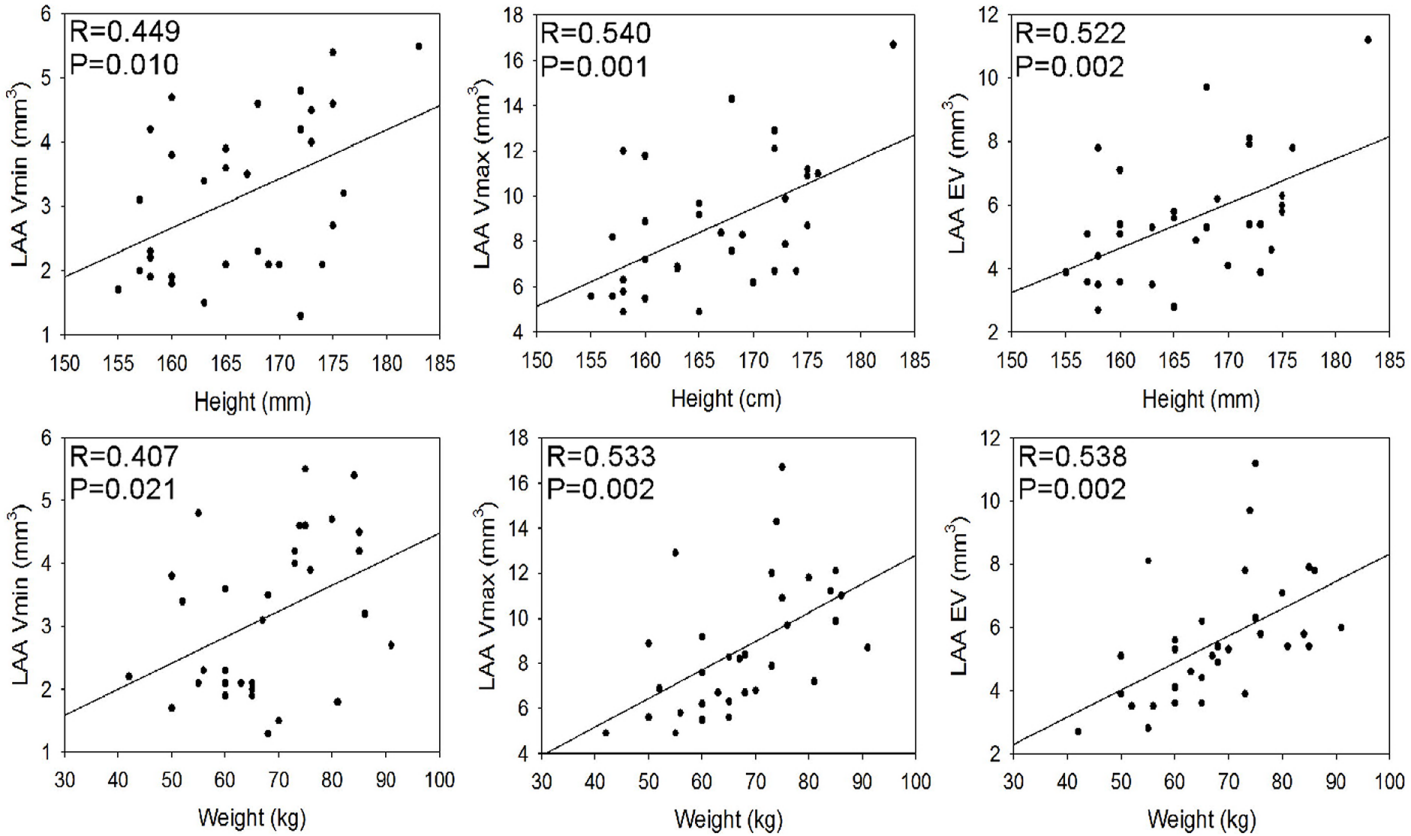
Correlation of the left atrial appendage (LAA) volume with the body height and weight. The body’s height and weight are significantly (P<0.05) positively correlated with the minimal, maximal and emptying volumes of the LAA. LAA Vmax, the LAA maximal volume; LAA Vmin, the LAA minimal volume; LAA EV, the LAA emptying volume.

**Fig 9:**
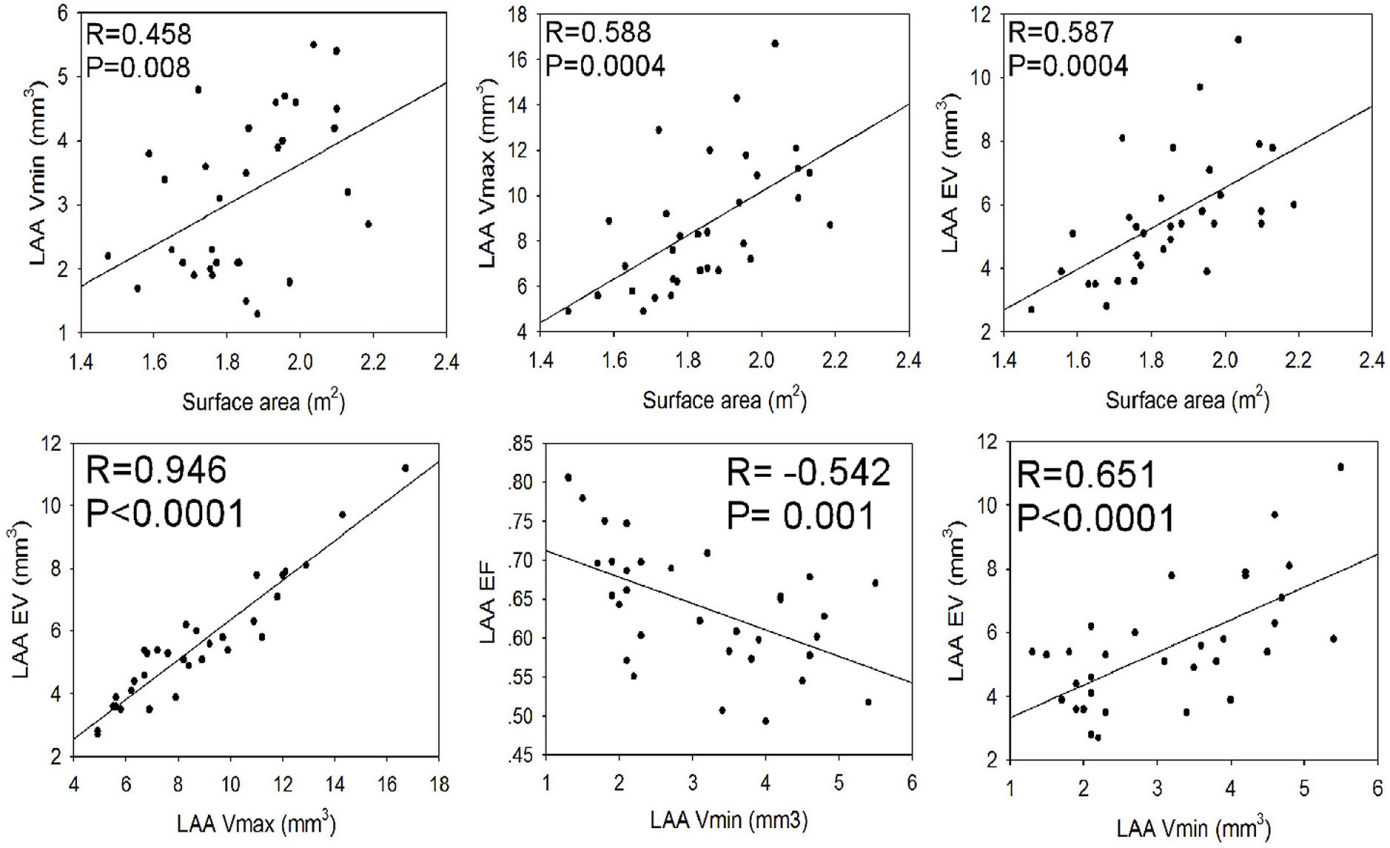
Correlation between the left atrial appendage (LAA) volumes and the body surface area. The body surface area is significantly positively correlated with the LAA volumes and emptying volume. The LAA maximal volume is positively correlated with the emptying volumes of the LAA, and the LAA minimal volume is correlated negatively with the LAA ejection fraction but positively with the LAA emptying volume. LAAVmax, the LAA maximal volume; LAAVmin, the LAA minimal volume; LAAEF, the LAA ejection fraction; LAAEV, the LAA emptying volume.

The LAA volume was measured at 5%-95% phases in the cardiac cycle, and the LAA volume changed with different phases in the cardiac cycle. The greatest volume was present at 45% phase corresponding to the late diastolic stage of the LAA while the smallest volume was at 5% phase corresponding to the late systolic stage of the LAA (Fig 7). We drew the volume-phase curve of the LAA for the first time and found that the curvature had two alternate rises and drops, corresponding to two filling and two emptying processes, during one cardiac cycle (Fig 7). No statistically significant difference (P>0.05) was found in the LAA maximal and minimal volumes, ejection fraction and emptying volume in males versus females (Table 6). However, the maximal, minimal and emptying volumes of the LAA were all positively correlated with the body height, weight and surface area, whereas the LAA ejection fraction had an inverse correlation with the minimal volume of the LAA but no correlation with the maximal volume of the LAA (Figs 8 and 9).

**Table 6:** Comparison of left atrial appendage volume and function between genders.

	Range ($\bar{\text{x}}$±s, *n* = 40)	Males (*n* = 20)	Females (*n* = 20)	*P*
LAAVmax (ml)	4.9–16.7(8.71±2.90)	9.62±2.98	7.90±2.67	0.095
LAAVmin (ml)	1.3–5.5(3.15±1.23)	3.48±1.37	2.86±1.06	0.159
LAAEF (%)	49.4–80.6(63.91±7.75)	64.25±8.49	63.61±7.27	0.820
LAAEV (ml)	2.7–11.2(5.55±1.94)	6.14±1.97	5.04±1.82	0.112

Note: LAA, the left atrial appendage; LAAVmax, the maximal volume of the LAA; LAAVmin, the minimal volume of the LAA; LAAEF, the ejection fraction of the LAA; LAAEV, the emptying volume of the LAA.

## Discussion

For the treatment of atrial fibrillation during the last decade, drug therapy has been accompanied by several interventional approaches as circumferential pulmonary vein isolation, roof and posterior wall left atrium linear lesions and complex fractionated atrial electrogram ablations[23]. To date, catheter-based circumferential pulmonary vein isolation has become a commonly performed procedure for symptomatic drug refractory atrial fibrillation[24], and trans-catheter device occlusion of the LAA has also emerged as an alternative treatment option to anticoagulation agents or surgery for prevention of thromboembolism originated from the LAA[25]. In these settings, precise characterization of the LAA, the left atrial structures, the pulmonary veins and the adjacent structures has become extremely important for successful operation and avoidance of intraprocedural complications[26]. In this study, we found substantial variability in the dimensions and anatomy of the LAA and its relationship to the adjacent structures. Given this great heterogeneity, a one-size-fits-all approach may not be optimal for all patients with respect to cardiac procedures including percutaneous catheter ablation for atrial fibrillation and trans-catheter device occlusion of the LAA.

In most literature, the LAA was described as “a long, tubular, hooked structure” of variable morphology and size[9, 13, 27–32]. However, our study detected substantially variable morphologies of the LAA. Lacomis et al [33] divided the LAA anatomical shape into 3 types (types 1, 2 and 3 corresponding to type 1a, type 2a and type 3, respectively, in our classification) based on the orientation and location of the LAA tip in cardiac CT angiography in 2007. These authors used a 64-detector scanner for cardiac CT angiography, however, their patient cohort was too small with only 45 patients enrolled, which probably accounts for fewer types in their LAA classification. In 2012, Koplay et al [28] recruited 320 patients in their MDCT coronary angiography by a 64-slice CT scanner and expanded the LAA shape into 5 types (type 1: horseshoe, type 2a: hand-finger, type 2b: paper fan, type 2c: wing, type 3: hook, type 4: wedge and type 5: swan), with 3 subtypes in the second type. In our study enrolling 670 patients in the MDCT coronary angiography with a 256-slice CT scanner, 2 new types (type 6: fork and type 7: ring) and 3 subtypes (type 1a: horseshoe, type 1b: cockscomb and type 2d: mountain) were identified besides the 5 types defined by Koplay et al [28]. Another classification of the LAA morphology was made by Wang et al [34] in 2010, consisting of 4 types: chicken wing, windsock, cauliflower and cactus according to the bend and lobes of LAA of 64-channel cardiac angiography. However, this classification is too simple and may have a major flaw in that they did not take into consideration the orientation and location of the LAA tip which is relatively fixed at different phases of the cardiac cycle[33]. Moreover, some types may be overlapped with each other (for example, the Cactus type may look like the Windsock type from the figures provided by the authors[34]). The authors of this classification [34] just used a very simple principle to classify the LAA morphology: LAA with a sharp bend or not. If the LAA had a sharp bend, it would be called the chicken wing type; if the LAA did not have a sharp bend, it would be divided into three types: windsock, cauliflower and cactus. This classification just took into consideration the mere shape of the LAA without looking into the anatomy, especially the location and orientation of the LAA tip. Our study investigated 670 patients and made a more detailed classification of the LAA morphology based on its shape and anatomy, especially the location and orientation of the LAA tip, thus being more practical. In order to install an occluding device to the LAA, the cardiac intervention therapist needs to puncture the atrial septum to send the device from the right atrium to the left atrium for occluding the LAA orifice, and the exact information of the location and orientation of the LAA can be greatly helpful in guiding the puncture and increase the success rate of the device installation. Even though some types may only account for a small portion (1.3% for type 1b and 0.7% for type 7), this cohort of study has only hundreds of people. However, in the real world with a population of billions of people, the number of people with these two small-portion types may be incredibly great! Thus, even the 7 types and 6 subtypes may not be sufficient to cover all the types of the LAA morphology in the world, which is a great alert for surgeons who are involved in the endovascular cardiac procedures. The real world is extremely complicated, thus necessitating a complicated classification system to deal with a complex structure like the LAA.

Whether the morphology of LAA is associated with nationality is not clear. Among the four studies categorizing the LAA anatomical shape including ours, two studies[33, 34] were from the United States of multinationalities, one study was from Turkey[28], and our study was from China. Turkey is a country straddling the Eurasia, and only China is located in Asia. However, our classification of the Chinese LAA morphology is similar to that from the Turkish study with 5 types and 3 subtypes in the Turkish people[28]. This may indicate that the LAA morphology is not associated with nationality of the patients and that our results may be applied to other nationalities.

In selection of the proper size of a LAA occluding device in percutaneous LAA transcatheter occlusion procedure, it is critical to know the morphology and size of the LAA orifice. Early clinical experience of percutaneous LAA occlusion demonstrated that the actual size of the selected occluding devices was frequently 20%-40% larger in diameter than predicted by transesophageal echocardiography[35]. One study revealed that successful implantation may need 1–4 attempts to choose a suitable LAA occluding device matching the size of the LAA [22], and additional manipulation to replace the occluding device will undoubtedly increase the procedural risks. In this study, the LAA morphology parameters did not correlate with age, indicating the LAA size in normal adults does not change with age. However, the LAA orifice had a significantly greater (P<0.01) long axis in men than in women, indicating the LAA orifice is more elliptical in men. Moreover, the LAA orifice long axis was positively correlated with the height, weight and surface area of the patient while the orifice short axis negatively with the above parameters, which may suggest that the taller an individual, the longer the long axis but the shorter the short axis of the LAA orifice, resulting in more elliptical of the LAA orifice in taller people. The LAA morphology parameters (orifice short and long axes, perimeter, area, and the LAA volume) displayed strong positive correlation with the left atrium volume, aortic cross area long axis or LSPV long axis but poor correlation with the height, weight, surface area and vertebral body height of the patients (Fig 3), suggesting that the LAA is closely related to the development of the heart and adjacent structures rather than the body statue.

The LAA is closely associated with many cardiac diseases and thromboembolic events, however, up to date, the normal reference range of the LAA parameters has not been defined. Ernst et al [36] studied the cast of the LAA in 220 autopsies and reported the range of the LAA parameters as follows: LAA volume 770–19270 (mean 5220±3041) mm^3^, orifice 5–27 (15±4) mm, and length 16–51 (mean30±5) mm. However, their study subjects were only corpses in whom the LAA may have shrunk in the process of preservation, incomparable to normal living people. Moreover, their subjects had various heart diseases and did not truly represent normal living people. The subjects in our study were all normal people without any cardiac diseases and the values of the LAA parameters can really represent the 95% reference range of normal living people (Table 3).

Recently, the LAA has been reported to be an underrecognized trigger site of atrial fibrillation and responsible for arrhythmias in 27% of patients presenting for repeat ablation procedure[20], indicating that the LAA is an additional ablation site. In this setting, precise evaluation or visualization of the left atrium, LAA and pulmonary anatomy is mandatory for complete ablation and prevention of possible complications. The very close vicinity of the LAA orifice with the proximal left circumflex coronary artery (2.1±0.9 mm, range: 1.0–6.6 mm in this study) requires the ablation near the anterior base of the LAA to be carried out with great caution to avoid potential risk of the left circumflex coronary artery injury (Fig 6). Nguyen-Do et al [37] had reported a case of fistula formation between the left circumflex coronary artery and the left atrium as a complication of radiofrequency cardio-ablation. When performing catheter ablation, the surgeons had better understand the LAA ridge width, length, morphology and the pulmonary vein orifice so that they could effectively control the catheter route of ablation for prevention of possible complications like pulmonary vein stenosis. Moreover, the width of the LAA ridge may affect the safety of LAA device occlusion. For example, the deployment of a LAA occlusion device may markedly compress the left pulmonary vein when the LAA orifice is very flat and in close proximity to the left pulmonary vein.

The LAA ridge was previously classified into two types [21]: type A (the ridge extends from the superior portion of the LSPV orifice to the inferior portion of the left inferior pulmonary vein (LIPV) orifice, Fig 5 Types A I and A II) and B (the ridge extends from the superior portion of the LSPV orifice to the intervenous saddle between the LSPV and LIPV orifices, Fig 5 Type B). However, we found a new type and named it as type C, in which the LSPV and the LIPV has a common venous trunk and the LAA ridge runs from the upper edge of the pulmonary common trunk to its lower edge (Fig 5 Type C). Besides, based on whether the LAA ridge was on the same plane with the intervenous saddle between the LSPV and the LIPV, we divided the LAA ridge into type I (the LAA ridge is on the same plane with the intervenous saddle between the LSPV and LIPV, Fig 5 Type A I) and type II (the LAA ridge is markedly prominent above the intervenous saddle between the LSPV and LIPV, Fig 5 Type A II).

The LAA body contains trabeculated pectinate musculature and is actively contractile in the hearts of healthy young people with normal intracardiac conduction[38]. Its filling and emptying is complex and greatly influenced by contraction, dilatation and distortion of the heart especially the left ventricle [9]. It adjusts the hemodynamics of the left atrium when the left atrial pressure and volume increase[30]. The LAA oscillates faster than 300 cycles/min in atrial fibrillation and its outflow velocities are reduced from approximately 40 cm/s during normal sinus rhythm to less than 20 cm/s in atrial fibrillation. The LAA emptying can be reduced to negligible levels in persistent atrial fibrillation, resulting in stasis, enlargement of the LAA to 2–3 times its normal volume, and thrombosis which is considered a prime cause of embolic cerebral infarction [39, 40]. The size and volume of LAA can be affected by many factors, and atrial fibrillation, left ventricular hypertrophy and/or myocardial scars can all increase the LAA size and volume [36]. The left ventricular function is also closely related to the LAA mechanical function, and decrease of the left ventricular ejection fraction will lead to decreased LAA function but increased cardiac thrombosis rate. Therefore, accurate evaluation of the LAA volume and function is critical to clinical prevention, risk stratification, diagnosis and therapeutic guidance of atrial fibrillation and other cardiac diseases.

This study confirmed that the LAA have two filling and two emptying processes during one cardiac cycle, which is consistent with the quadriphasic appendage flow reported by others[9, 41, 42]. During the 0%-45% phases, the curvature increased continuously sharply, indicating the first filling stage of the LAA with fast volume expansion. The 45%-65% phases corresponded to the first decrease of the curvature, indicating the first emptying of the LAA. The LAA was then filled again during the 65%-75% phases, but with slow volume expansion possibly caused by elastic recoil of the LAA. Later, the curvature had a sharp drop during the phases 75%-95%, indicating the second but fierce emptying of the LAA possibly caused by active contraction of the LAA. During the 65%-85% phases, the fluctuation magnitude of the curvature was not great and the LAA volume remained relatively constant as if the LAA was “at rest” (Fig 7). The measurement of the LAA volume, orifice axis and cross area at this stage would be closer to the “true value”. The positive correlation of the LAA maximal, minimal and emptying volumes with the body height, weight and surface area indicated that both the LAA volume and output rise with increase of the body height, weight and surface area.

Echocardiography, CT, magnetic resonance angiography and conventional angiography have all been used to evaluate the LAA. Recent CT technological advances such as multidetector scanners make it possible to visualize the cardiac anatomy and vascular structures in detail. MDCTA was introduced in 1999 and since then has steadily increased its application in the cardiovascular clinical arena, from the initial use of coronary angiography to the latest left ventricular function analysis, stress myocardial perfusion, detection of myocardial fibrosis and viability, and detailed surface structures like the LAA and pulmonary veins. Moreover, newer generation of MDCT scanners have dramatically improved spatial resolution to a maximum of 0.35 mm isotropic voxel size (0.35 mm in each side) [43]. MDCT is a reliable and noninvasive tool for diagnosing vascular anomalies and in the determination of cardiac anatomy. The combination of MDCT with new 3D reconstruction software allows direct accurate measurement of left atrial, ventricular and LAA surface and volume without geometrical assumptions[44, 45]. Published data on the use of magnetic resonance angiography and CTA for evaluating the LAA have focused primarily on the identification of LAA thrombus. In this study, we provided a detailed evaluation with 256-slice CTA documenting significant interpersonal variability in LAA size and morphology from a series of people and provide the normal range of values of the LAA size and morphology. Given an increasing interest in trans-catheter device occlusion of LAA and percutaneous catheter ablation for atrial fibrillation, the normal range of the LAA size and morphology and the LAA relationship with the adjacent structures is very important in guiding appropriate selecting and sizing of occlusion devices and sites of catheter ablation.

Our study has several important findings. Firstly, contrary to the common image of the LAA described in most literature, our study revealed that the LAA was absolutely not “a long, tubular, hooked structure” of variable morphology and size [9, 13, 27–32]. Among the 7 types and 6 subtypes in our classification, only type 1a, type 3 and type 4 have the morphology similar to “a long, tubular, hooked structure”, and the most prevalent type (type 2) is anything but “a long, tubular, hooked structure”. Moreover, there are quite some small lobes in addition to some larger ones attached to the LAA in every type, making it more cauliflower-like. Secondly, the substantial variability of the LAA size and morphology regarding the LAA volume, orifice dimensions, depth, morphology and its relationship with adjacent structures such as the left pulmonary veins found in this study may help planning for trans-catheter LAA device closure and catheter ablation. Thirdly, we set up the normal range of the values of the LAA parameters (LAA size, morphology, relationship with the adjacent structures) and the LAA ridge width and length, this normal range is lacking in the current literature. As stated before, the LAA orifice is related to the body statue of the patient. In men and people with a taller and bigger statue, the LAA orifice is more elliptical, thus beneficial to the selection of LAA occlusion device and catheter ablation site. Fourthly, the LAA ridge may not be on the same plane with the connection portion of the LSPV and LIPV. This may have very important clinical significance when performing trans-catheter ablation or device occlusion of the LAA. A detailed understanding of the LAA ridge may help preventing possible complications like pulmonary vein injury or stenosis. Fifth, the orifice long axis, minimal, maximal and emptying volumes of the LAA are all positively correlated with the body height, weight and surface area, indicating that both the LAA volume and emptying volume rise with increase of the body statue. This correlation helps guiding the trans-catheter ablation and device occlusion. However, since this study included only patients without atrial fibrillation, the results could be different in patients with atrial fibrillation.

In conclusion, the LAA has substantially variable morphology and relation with the adjacent structures, which may be helpful in guiding the LAA trans-catheter occlusion or catheter ablation procedures.
